# Pheochromocytoma and Hypertrophic Cardiomyopathy Leading to Cardiac Arrest

**DOI:** 10.7759/cureus.39986

**Published:** 2023-06-05

**Authors:** Kevyn Niu, Azhar Ghumra, Bilal Mirza, Jonathan Dreier

**Affiliations:** 1 Internal Medicine, Hospital Corporation of America (HCA) Florida Blake Hospital, Bradenton, USA; 2 Critical Care Medicine, Hospital Corporation of America (HCA) Florida Blake Hospital, Bradenton, USA

**Keywords:** catecholamines, ventricular fibrillation, hypertrophic cardiomyopathy, pheochromocytoma crisis, out of hospital cardiac arrest

## Abstract

A 33-year-old female with no known past medical history presented to the hospital for a witnessed cardiac arrest. The patient was emergently intubated and sedated. Further investigation demonstrated an 8.5 cm x 7.6 cm mass in the adrenal region, which was subsequently found to be a pheochromocytoma by biopsy. She was transferred to a tertiary care center for further evaluation. We wish to raise awareness of this condition among clinicians and encourage further research into the connections between pheochromocytoma and further cardiac complications.

## Introduction

Pheochromocytoma is a rare neuroendocrine tumor composed of chromaffin cells in the adrenal medulla. Symptoms of pheochromocytoma typically include hypertension, tachycardia, and diaphoresis, which are secondary to sympathetic nervous system hyperactivity as a result of a catecholaminergic surge [1]. This surge can result in cardiovascular complications, including cardiomyopathies [1]. Previous inquiries into this subject have described connections between the development of pheochromocytomas and various cardiomyopathies, such as Takotsubo and hypertrophic obstructive cardiomyopathy [2]. However, cardiac arrest from pheochromocytoma is exceedingly rare and poorly characterized [2]. We present the rare case of a 33-year-old female with no known medical history who experienced multiple cardiac arrests, ultimately attributed to a pheochromocytoma.

## Case presentation

A 33-year-old female with no known past medical history initially presented to the hospital for witnessed cardiac arrest from a nearby facility. Our patient received bystander cardiopulmonary resuscitation (CPR) for an unknown amount of time, as well as three rounds of defibrillation with an automated external defibrillator (AED). The initial rhythm type was unknown. Upon arrival at the hospital, the patient underwent emergent intubation with rapid sequence induction using etomidate and succinylcholine, followed by sedation with propofol.

**Figure 1:**
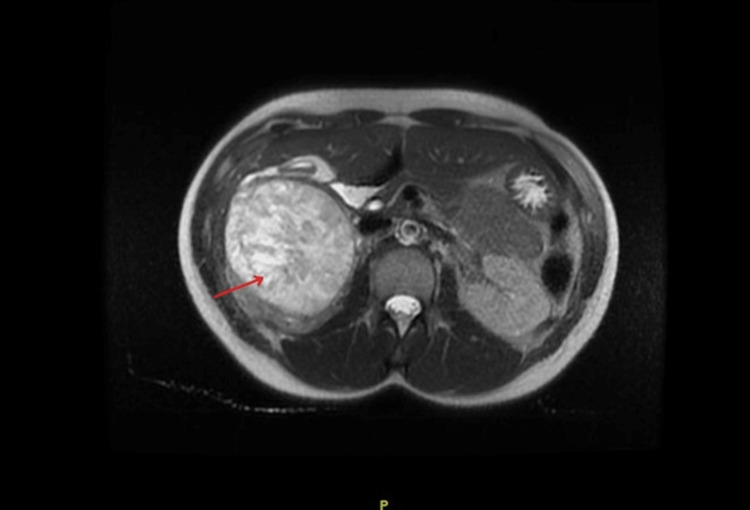
Axial T2-weighted MRI abdomen. The red arrow demonstrates a large 8 cm x 10 cm mass with heterogeneous high signal intensity in the right suprarenal/adrenal region. MRI: magnetic resonance imaging

Initial vital signs were significant for fever (100.4°F), tachycardia (123 bpm), and hypertension (165/93 mmHg). The initial 12-lead electrocardiogram (EKG) demonstrated sinus tachycardia, with possible depressions in the anterior leads. Initial laboratories were significant for leukocytosis (25,000 WBC/mm^3^), and lactic acidosis (3.3 mmol/L). The initial high-sensitivity troponin was elevated at 165 ng/L. Our patient received computed tomography (CT) imaging of the brain, C-spine, and face, which were negative for any acute findings. CT imaging of the chest demonstrated bilateral airspace consolidations with ground-glass opacities, concerning edema versus hemorrhage. An additional partially visualized heterogeneous oval lesion in the adrenal/suprarenal region was noted, measuring 8.5 cm x 7.6 cm. Subsequent abdominal T2-weighted magnetic resonance imaging (MRI) demonstrated an 8.2 x 8.0 x 10.2 cm right adrenal/suprarenal mass (Figure 1). Our patient was emergently brought to the intensive care unit (ICU) at this time.

Following admission, our patient sustained a significant kidney injury, with creatinine increasing from 1.4 mg/dL to 4.2 mg/dL over the course of two days. Our patient was instituted on hemodialysis per Nephrology recommendations. An autoimmune workup, including antinuclear antibody, c-ANCA, p-ANCA, double-stranded DNA antibodies, and glomerular basement antibodies, were all within normal limits. Interventional Radiology was consulted, who performed a confirmatory adrenal biopsy. Immunohistochemistry studies on the adrenal mass demonstrated positive synaptophysin, chromogranin, and vimentin, with negative S100, results favoring pheochromocytoma. Plasma metanephrines were found to be 181.7 pg/mL, normetanephrine>10000 pg/mL, norepinephrine >8000 pg/mL, and epinephrine 500 pg/mL, confirming our diagnosis of pheochromocytoma.

**Figure 2:**
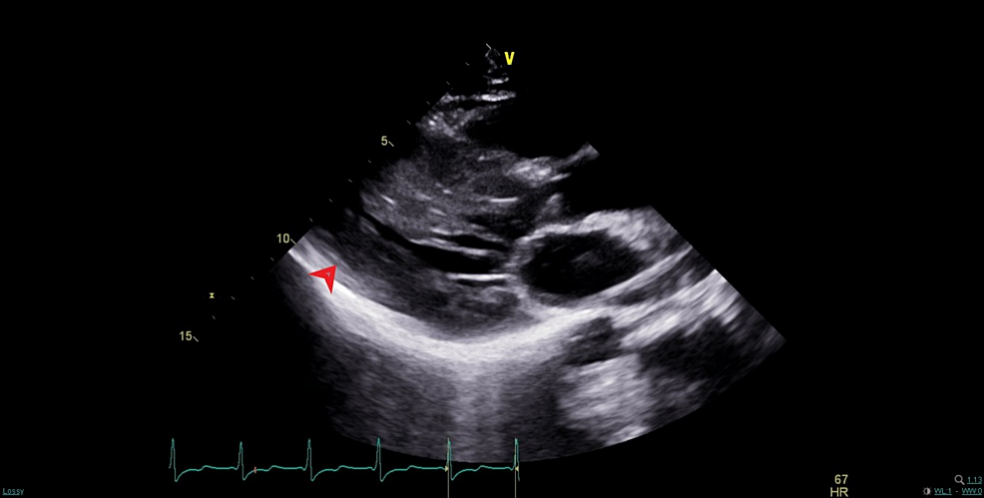
Transthoracic echocardiogram, parasternal long-axis view. The red arrow demonstrates significant left ventricular wall thickening, suggestive of hypertrophic cardiomyopathy.

Cardiology was consulted, who performed a transthoracic echocardiogram (TTE), which demonstrated an ejection fraction of 55-60%, remarkable for severe left ventricular hypertrophy with intraventricular septum measuring 17.1 mm suspicious for hypertrophic cardiomyopathy (Figure 2). A left heart catheterization demonstrated no coronary occlusions, with irregularities of less than 20% observed in all visualized vessels.

On day 20 of hospitalization, the patient went into cardiac arrest from polymorphic ventricular tachycardia, requiring three rounds of epinephrine, 300 mg of amiodarone, 2 g of magnesium, one round of defibrillation, and 50 cc 8.4% sodium bicarbonate. Return of spontaneous circulation (ROSC) was obtained around 20 minutes thereafter. Our patient remained stable for the remainder of her hospitalization. Shortly thereafter, our patient was transferred to a tertiary care center for pheochromocytoma resection, endomyocardial biopsy (EMB), and placement of an automated implantable cardioverter/defibrillator (AICD) for ventricular tachycardia.

## Discussion

Pheochromocytomas are rare and potentially deadly neuroendocrine tumors that arise from the chromaffin cells of the adrenal medulla and produce catecholamines [3]. The term pheochromocytoma is used specifically for sympathetic intra-adrenal paragangliomas [4]. They cause life-threatening systemic effects that manifest primarily as intermittent uncontrolled hypertension, tachycardia, and headaches [5]. The diagnosis of pheochromocytoma is based on high clinical suspicion and prompt biochemical testing to identify excess catecholamines and metanephrines in blood and urine samples [6]. Although the incidence is low, patients with uncontrolled hypertension are at greater risk of developing pheochromocytoma-induced cardiac dysfunction due to prolonged exposure to excess catecholamines [7]. This case highlights the importance of early identification and appropriate management of pheochromocytoma to prevent such complications.

Our patient presented with cardiac arrest and was persistently tachycardic and hypertensive throughout the hospital course. Later, it was discovered that she had significantly increased left ventricular wall thickness, which is most likely explained by the long-term systemic effects of her untreated pheochromocytoma.

A review of existing literature demonstrates several connections between pheochromocytoma and arrhythmias, cardiomyopathies, and cardiac arrest [8,9]. The exact mechanism behind pheochromocytoma-induced cardiomyopathy is not fully understood; however, it is believed that excess catecholamines produced by pheochromocytoma can over-stimulate cardiac myocytes, causing myocardial damage, oxidative stress, fibrosis, and hypertrophy that eventually manifest as various types of cardiac dysfunction [10]. Confirmation of hypertrophic cardiomyopathy is performed via left ventricular EMB. EMB is essential to differentiate patients with true hypertrophic cardiomyopathy from those with other causes of myocardial thickening, including cardiac amyloidosis. Our patient was transferred to a tertiary care center for evaluation with EMB.

Subsequent cardiac arrest resulting from catecholamine-induced cardiomyopathy is rare [11]. The role of excess catecholamines in sudden cardiac death is implicated in the pathogenesis of both pheochromocytoma-induced cardiomyopathy and Takotsubo cardiomyopathy [12]. The mechanism appears to be related to the action of alpha-adrenergic receptors in coronary arteries, causing vasospasm and subsequent cardiac ischemia. It is proposed that there may also be a role in oxidative stress and the production of oxyradicals, which cause cardiotoxic effects [12]. Similar mechanisms of myocardial injury have also been proposed in chronic cocaine use, and both cocaine-induced and pheochromocytoma-induced cardiac injury share a similar histological appearance [13]. Links to nodal dysfunction have also been explored - among hyper-catecholaminergic states, catecholamine-induced tachyarrhythmias that progress to ventricular fibrillation are the primary cause of cardiac arrest. Catecholamine-induced polymorphic tachycardia (CPVT) has been associated with diastolic calcium overload and subsequent sinus node dysfunction [14]. The treatment approach for CPVT is similar to that of other forms of ventricular tachycardia, involving the use of beta-blockers and calcium channel blockers [14].

Our patient is unique in that she experienced multiple cardiac arrests from the pheochromocytoma, including one in-hospital which was identified as ventricular tachycardia-induced. Paulin et al. suggest that the excess catecholamine levels can lead to abnormal myocardial electrical activity, causing massive ion influx resulting in myocardial auto-rhythmicity. In addition, these catecholamine levels can reduce the cardiac ventricular fibrillation threshold, predisposing to ventricular fibrillation and cardiac arrest [15]. Khan describes a similar case of treatment-resistant ventricular tachycardia requiring four direct current cardioversion shocks to terminate the VT. In that case, left ventricular non-compaction, a rare congenital cardiomyopathy leading to intertrabecular recesses in the myocardium, was found to be a contributing factor to cardiac arrest [16]. Our patient had multiple episodes of cardiac arrest, requiring three rounds of defibrillation the first time and one round during the second occurrence.

The management of pheochromocytoma-induced cardiomyopathy involves both medical and surgical approaches; once the diagnosis is confirmed, the primary treatment for pheochromocytoma is excision of the tumor [17]. This requires pre-treatment with an alpha-blocker before initiating beta-blocker therapy [17]. If a patient with heart failure has a concurrent pheochromocytoma and a beta-blocker is started before an alpha-blocker, this can result in adverse systemic effects, including paradoxical hypertension due to unopposed alpha-adrenergic activity [18]. Thus, the coexistence of cardiac dysfunction makes medical management more challenging since beta-blockers are part of the guideline-directed medical therapy for heart failure [19].

## Conclusions

Pheochromocytoma-induced cardiomyopathy leading to cardiac arrest is a rare phenomenon that is poorly understood in the literature. Our patient presents an uncommon case of cardiac arrest in a patient with no known medical history, who was found to have a pheochromocytoma. Our aim is to raise awareness of this condition among clinicians and encourage further research into the connections between pheochromocytoma, cardiac arrest, and further cardiac complications.
